# Modular brain networks shape amyloid‐driven tau spread and cognitive decline

**DOI:** 10.1002/alz.71617

**Published:** 2026-07-08

**Authors:** Fabian Hirsch, Lukas Frontzkowski, Anna Steward, Sebastian N. Roemer‐Cassiano, Davina Biel, Zeyu Zhu, Carla Palleis, Johannes Gnörich, Madleen Klonowski, Günter Höglinger, Matthias Brendel, Nicolai Franzmeier

**Affiliations:** ^1^ Institute for Stroke and Dementia Research (ISD) University Hospital, LMU Munich Munich Germany; ^2^ Department of Nuclear Medicine University Hospital, LMU Munich Munich Germany; ^3^ Department of Neurology University Hospital, LMU Munich Munich Germany; ^4^ Max Planck School of Cognition Leipzig Germany; ^5^ Department of Clinical Neurosciences University of Cambridge School of Clinical Medicine Cambridge UK; ^6^ German Center for Neurodegenerative Diseases (DZNE) Munich Germany; ^7^ Munich Cluster for Systems Neurology (SyNergy) Munich Germany; ^8^ Institute of Neuroscience and Physiology, Department of Psychiatry and Neurochemistry University of Gothenburg, The Sahlgrenska Academy Gothenburg Sweden

**Keywords:** graph theory, integration, neurodegeneration, neuroimaging, segregation, tauopathy

## Abstract

**INTRODUCTION:**

Alzheimer's disease involves trans‐synaptic spread of tau pathology from temporal lobe epicenters, driven by amyloid beta (Aβ) deposition. How modular brain network architecture shapes this process and the ensuing cognitive decline remains incompletely understood. We tested whether the efficiency with which tau epicenters access cross‐network communication pathways modulates Aβ‐driven tau propagation.

**METHODS:**

We combined baseline/longitudinal amyloid/tau positron emission tomography (PET) data across two independent AD cohorts (*N *= 490) with multimodal connectomics data. We quantified epicenter broadcast capacity (EBC), capturing whether tau epicenters preferentially access regions supporting cross‐network communication or within‐network communication.

**RESULTS:**

Higher EBC was associated with faster Aβ‐related global tau accumulation, greater spatial tau spread, and steeper cognitive decline. Effects were driven by stronger epicenter communication with cross‐network connectors, whereas preferential within‐network routing was associated with relative containment of tau spread.

**DISCUSSION:**

We identify a mechanism through which epicenter connectivity biases Aβ‐driven tau propagation toward brain‐wide broadcast or regional containment, helping explain heterogeneity in disease progression.

## Highlights

- Network embedding of tau epicenters shapes amyloid‐driven tau spread in AD.
- Distinct epicenter communication pathways moderate amyloid‐related pathology.
- Efficient communication with cross‐network connectors accelerates tau spread.
- Efficient within‐network communication limits tau spread and cognitive decline.

## 1 BACKGROUND

AD is defined by a cascade of pathological brain changes culminating in neurodegeneration, cognitive decline, and dementia.^1^, ^2^ The temporal sequence of neuropathological changes preceding symptom manifestation forms the basis of the amyloid cascade model of AD,^3^, ^4^ positing that cortical Aβ deposition initiates the aggregation and spread of neurofibrillary tau pathology typically from temporal lobe epicenters to the neocortex,^5^, ^6^ which in turn drives neurodegeneration and cognitive decline.^7^, ^8^, ^9^ Preclinical research has shown that tau spreads trans‐synaptically in an activity‐dependent manner, with neuronal connections acting as a scaffold for tau spread throughout the brain.^6^, ^10^ Further, Aβ is a potent driver of neuronal hyperactivity,^11^, ^12^ which may specifically amplify the synaptic release of hyperphosphorylated tau seeds,^13^, ^14^ thereby facilitating trans‐synaptic tau spread.^6^, ^15^ We and others previously translated these concepts to in vivo neuroimaging data, combining positron emission tomography (PET) of fibrillar amyloid beta (Aβ) and tau pathology with structural and functional connectomics to determine inter‐regional connectivity. Results corroborate that tau spreads from regions with high baseline tau burden (i.e., tau epicenters) across connected regions^16^, ^17^, ^18^, ^19^ and that Aβ induces phosphorylated tau (p‐tau) secretion and neuronal hyperconnectivity, thereby promoting tau spread.^20^, ^21^ These findings support a mechanistic model in which both brain network topology and Aβ‐induced neuronal connectivity changes contribute to the spread of tau in AD.^20^, ^22^, ^23^ Further elucidating the specific role of brain networks in tau spread is important for understanding the heterogeneity in tau deposition patterns,^18^, ^24^ tau aggregation speed,^25^ and downstream clinical trajectories observed across the AD spectrum.^7^, ^26^

A key feature of brain networks that may influence the dynamics of Aβ‐driven tau spread in AD is modularity,^27^, ^28^ which refers to the organization of brain regions into specialized modules with dense intramodular connectivity and sparse intermodular connections.^27^, ^29^ Within this modular architecture, some regions preferentially interconnect otherwise segregated brain systems via cross‐module links (“connectors”), whereas others mainly communicate within their own functional modules (“local nodes”).^30^ Supporting a role of these network properties in tau aggregation dynamics, we found previously that lower modularity was linked to faster tau spread from epicenters to connected regions^31^ and stronger cognitive deficits in AD,^32^ possibly since a less modular structure would facilitate tau spread across module boundaries and, therefore, cognitive decline.^33^ Further, we reported that the emergence of tau in globally connected brain regions was associated with faster subsequent tau accumulation and earlier AD symptom onset.^25^ These findings support the view that the brain's modular network architecture influences the rate and pattern of tau spread. However, it is unclear whether Aβ‐driven tau propagation from individual tau epicenters is shaped by their communication efficiency to connectors versus local nodes along axonal pathways. Addressing this distinction is pivotal, as local nodes could constrain tau dissemination mainly within functional modules, while connectors may facilitate the network‐wide broadcast of tau across diverse cortical systems, accelerating overall AD progression.

**RESEARCH IN CONTEXT**

1. **Systematic review**: The authors reviewed the literature on Aβ‐associated tau propagation in AD. Prior work suggested that tau spreads trans‐synaptically from localized tau epicenters, a process promoted by Aβ. It remains unclear how the brain's organization into largely segregated but interacting functional networks shapes Aβ‐driven tau propagation toward brain‐wide dissemination or regional containment.
2. **Interpretation**: Our findings extend existing network‐based models of AD by suggesting that communication patterns between tau epicenters and regions with distinct system‐level network embedding (cross‐network vs. within‐network) shape Aβ‐driven tau dynamics and clinical progression across the AD spectrum.
3. **Future directions**: Future studies could determine whether regions that facilitate cross‐network communication represent key nodes for network‐informed therapeutic targeting. Testing whether modulation of these propagation pathways can limit large‐scale tau dissemination may provide new strategies for slowing AD progression and inform more personalized intervention approaches in clinical practice.

**FIGURE 1:**
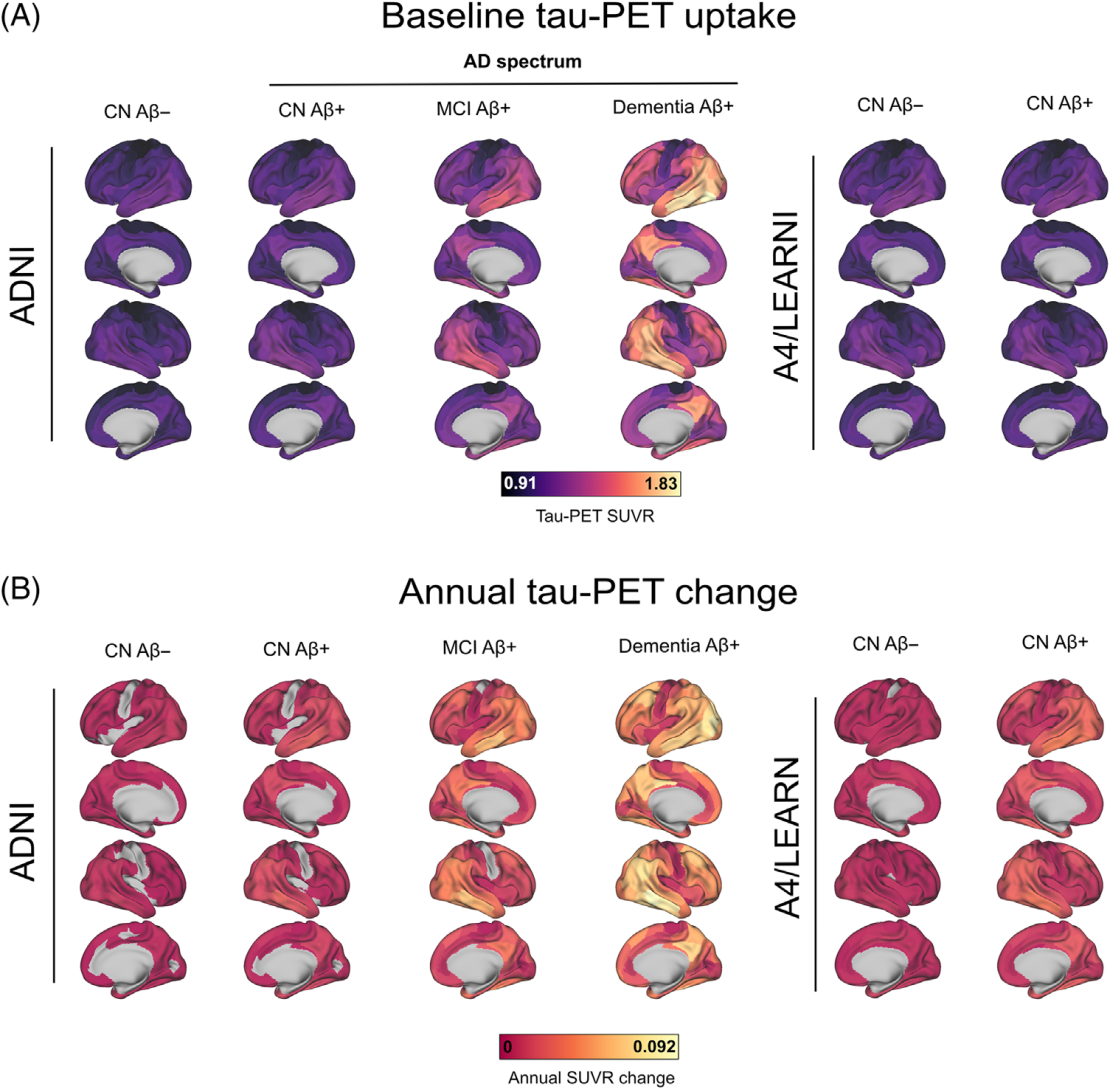
Average baseline tau‐PET uptake and longitudinal tau‐PET changes for ADNI and A4/LEARN samples. (A) Surface projections of average baseline tau‐PET SUVR values stratified by diagnostic group and Aβ status in ADNI (left), showing increasing tau load across the AD spectrum. Corresponding values for Aβ‐stratified A4/LEARN subjects (right). (B) Surface projections of average annual tau‐PET SUVR changes, stratified by diagnostic group and Aβ status at baseline in ADNI (left), illustrating faster tau accumulation in Aβ+ patients (AD spectrum). Corresponding values for Aβ‐stratified A4/LEARN subjects (right). Aβ,  amyloid beta; AD,  Alzheimer's disease; CN,  cognitively normal; MCI,  mild cognitive impairment; Roc,  rate of change; SUVR,  standardized uptake value ratio.

**FIGURE 2:**
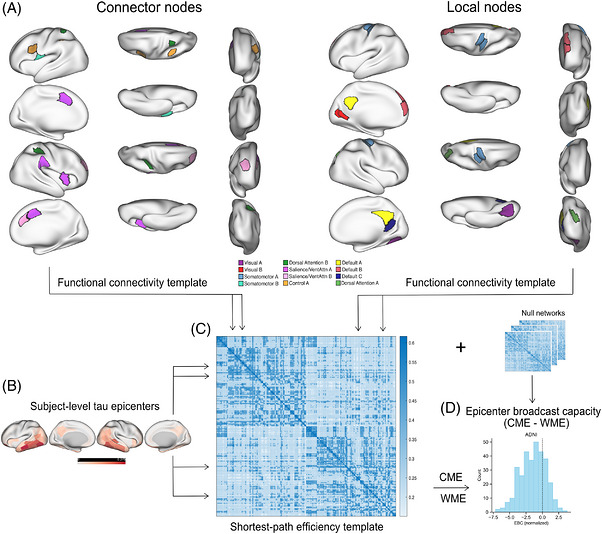
Calculation of epicenter broadcast capacity (EBC). (A) Spatial distribution of functional connector and local regions derived from a resting‐state fMRI data functional connectivity template, color‐coded by canonical resting‐state networks. (B) Identification of subject‐level tau epicenters, defined as regions exhibiting the highest baseline tau‐PET uptake. (C) Estimation of epicenter‐node communication using a shortest‐path efficiency template derived from diffusion MRI, yielding average epicenter‐based efficiency to connectors (CME) and local nodes (WME). (D) EBC is defined as the difference between CME and WME, normalized relative to a distribution of matched null networks.

**FIGURE 3:**
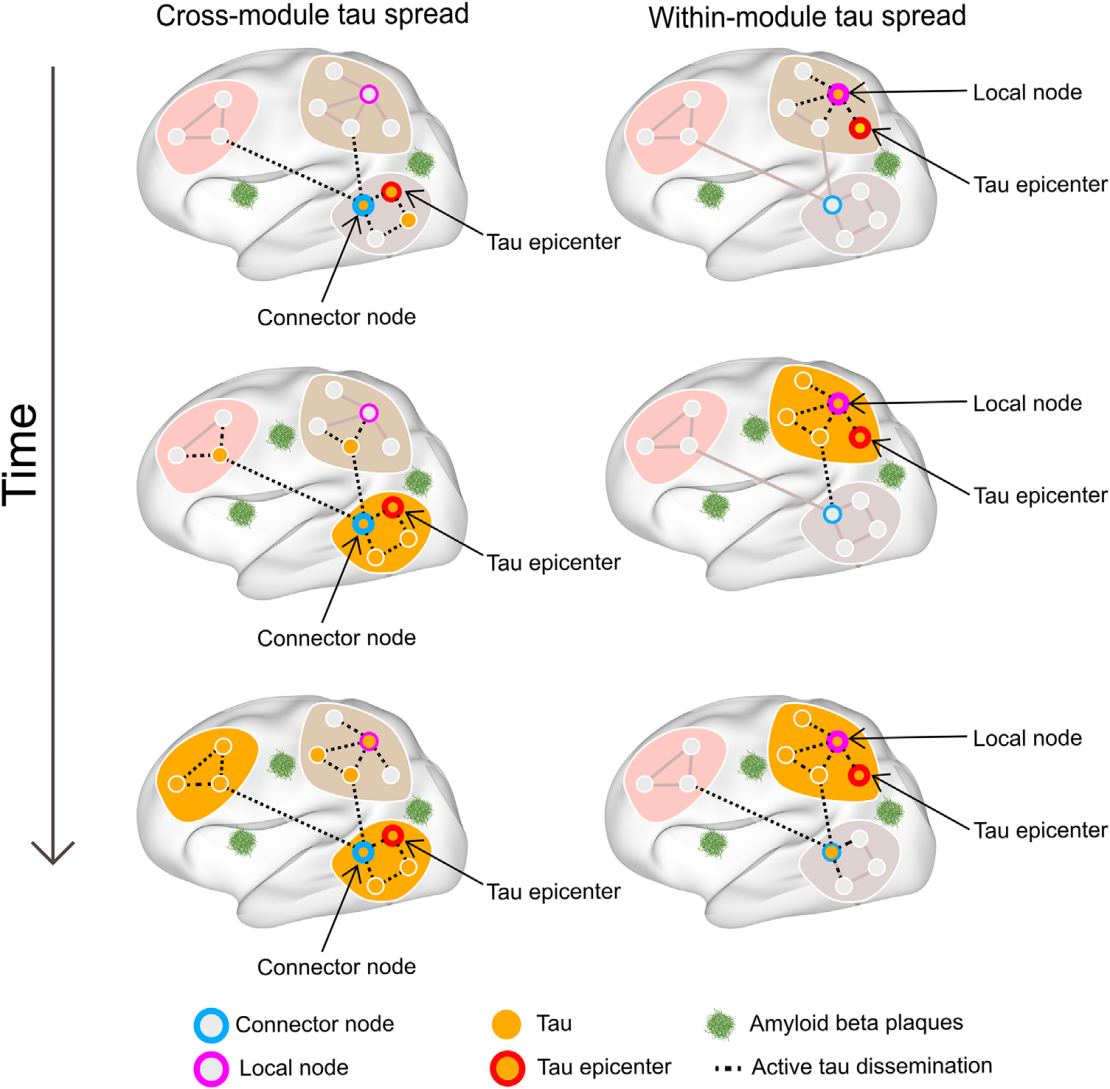
Simplified schematic showing hypothesized effects of network topology on connectivity‐mediated tau spread in AD, starting from initial tau epicenters. More efficient network routing of tau epicenters to connector regions (left column) leads to more widely distributed tau pathology spread across different functional modules as the disease progresses over time. In contrast, more efficient network routing of tau epicenters to more locally connected regions (right column) is associated with more localized within‐module tau pathology spread as the disease progresses over time. AD, Alzheimer's disease.

Thus, the main aim of the current study was to test whether the propensity of individual tau epicenters to communicate more efficiently with connectors than local nodes would amplify the impact of baseline Aβ on subsequent tau spread and cognitive decline. We addressed this question in two cohorts comprising 490 individuals across the AD spectrum with available amyloid‐PET and longitudinal tau‐PET data (Figure 1). Using template‐based connectomics, we quantified the communication efficiency between patient‐specific tau epicenters and connector (cross‐module efficiency [CME]) and local nodes (within‐module efficiency [WME]), using functional connectivity for node classification and structural connectivity for estimating communication pathways (Figure 2; see “Methods”). We subsequently defined epicenter broadcast capacity (EBC) as the difference between CME and WME, capturing whether tau epicenters preferentially communicated with connectors rather than local nodes (Figures 2D and 3). We then tested whether higher EBC amplified Aβ‐related effects on (i) global tau accumulation rates, (ii) downstream tau deposition across functional systems, and (iii) cognitive decline.

## 2 METHODS

### 2.1 ADNI sample

As the primary (discovery) dataset, we included 325 individuals from ADNI with available baseline [^18^F]florbetapir/florbetaben amyloid‐PET and longitudinal [^18^F]flortaucipir tau‐PET data, including patients across the AD spectrum (cognitively normal [CN]/Aβ negative [Aβ−]: *n *= 123; CN/Aβ‐positive [Aβ+]: *n *= 99; mild cognitive impairment [MCI]/Aβ+: *n *= 70; Dementia/Aβ+: *n *= 33; Table 1). Baseline data were obtained within a time frame of 6 months, and diagnostic status was determined by ADNI as CN (Mini‐Mental State Examination [MMSE] ≥ 24, Clinical Dementia Rating [CDR] = 0, non‐depressed), MCI (MMSE ≥ 24, CDR = 0.5, objective memory impairment on education‐adjusted Wechsler Memory Scale II, preserved activities of daily living), and demented (MMSE = 20 to 26, CDR > 0.5, National Institute of Neurological Disorders and Stroke/Alzheimer's Disease and Related Disorders Association criteria for probable AD). Amyloid status was determined by global amyloid‐PET SUVRs via tracer‐specific cut‐offs of 1.11/1.08 for florbetapir/florbetaben, as previously established in the ADNI cohort.^34^, ^35^ All study procedures were conducted in accordance with the Declaration of Helsinki, with ethical approval obtained by ADNI investigators. All study participants provided written informed consent.

**TABLE 1 alz71617-tbl-0001:** Sample characteristics in ADNI.

ADNI (*N* = 325)	CN (Aβ−) *n* = 123	CN (Aβ+) *n* = 99	MCI (Aβ+) *n* = 70	Dementia (Aβ+) *n* = 33	*p* value
Age (M/SD)	72.36 (6.65)	74.86 (7.19)	74.89 (7.11)	76.13 (8.13)	0.067
Sex (male/female)	58/65	37/62	40/30	20/13	0.269
MMSE (M/SD)	29.15^[Link]^, ^[Link]^ (0.94)	28.96^[Link]^, ^[Link]^ (1.43)	27.41^[Link]^, ^[Link]^, ^[Link]^ (2.26)	21.89^[Link]^, ^[Link]^, ^[Link]^ (3.84)	<0.001
Centiloid (M/SD)	7.65^[Link]^, ^[Link]^, ^[Link]^ (8.42)	64.99^[Link]^, ^[Link]^, ^[Link]^ (33.61)	78.89^[Link]^, ^[Link]^, ^[Link]^ (29.53)	95.85^[Link]^, ^[Link]^, ^[Link]^ (31.32)	<0.001
Amyloid tracer (AV45/FBB)	89/34	74/25	44/26	17/16	0.393
Tau‐PET global SUVR (M/SD)	1.07^[Link]^, ^[Link]^ (0.08)	1.10^[Link]^, ^[Link]^ (0.09)	1.20^[Link]^, ^[Link]^, ^[Link]^ (0.20)	1.38^[Link]^, ^[Link]^, ^[Link]^ (0.38)	<0.001
Tau‐PET follow‐up years (M/SD)	3.70^[Link]^, ^[Link]^, ^[Link]^ (1.49)	2.85^[Link]^, ^[Link]^ (1.46)	2.52^[Link]^, ^[Link]^ (1.43)	1.75^[Link]^, ^[Link]^, ^[Link]^ (0.65)	<0.001

*Note*: Means (SD) are shown for continuous variables, counts for categorical variables. ANOVA tests were used for continuous variables, and chi‐squared tests were used for categorical variables in group comparisons. Post hoc pairwise comparisons were performed using Tukey's Honestly Significant Difference. *P* values are Bonferroni‐corrected for multiple comparisons.

^a^ *p* < 0.05 versus CN Aβ−.

^b^ *p* < 0.05 versus MCI Aβ+.

^c^ *p* < 0.05 versus CN Aβ+.

^d^ *p* < 0.05 versus Dementia Aβ+.

### 2.2 A4/LEARN sample

As an independent (validation) dataset we used data from 165 Aβ+ participants of the A4/LEARN study based on the availability of clinical/demographic data, baseline [^18^F]florbetapir amyloid‐PET, and longitudinal [^18^F]flortaucipir tau‐PET (Table 2). All participants were classified as CN (MMSE > 25, CDR = 0, Wechsler Logical Memory score of 6 to 18), as defined by the inclusion criteria of the A4/LEARN trial. Amyloid positivity was defined by a Centiloid scale threshold of at least 25 (see “Image processing”), aligning with tracer‐specific thresholds for amyloid status used in ADNI (see “ADNI sample”). All study procedures were conducted in accordance with the Declaration of Helsinki, and ethical approval was obtained by A4/LEARN investigators. All study participants provided written informed consent.

**TABLE 2 alz71617-tbl-0002:** Sample characteristics in A4/LEARN.

A4/LEARN (*N* = 165)	CN (Aβ+)
Age (M/SD)	71.62 (4.71)
Sex (male/female)	65/100
MMSE (M/SD)	28.61 (1.32)
Centiloid (M/SD)	64.89 (29.80)
Tau‐PET global SUVR (M/SD)	1.10 (0.09)
Tau‐PET follow‐up years (M/SD)	4.39 (1.40)

*Note*: Means and standard deviations are reported for continuous variables, absolute numbers for categorical measures.

### 2.3 Neuroimaging acquisition

Structural magnetic resonance imaging (MRI) and functional MRI (fMRI) were acquired using 3T Siemens, GE, and Philips scanners. T1‐weighted structural scans were collected with a MPRAGE sequence (repetition time = 2300 ms; voxel size = 1 × 1 × 1 mm). Resting‐state fMRI was performed via a two‐dimensional echo‐planar imaging (EPI) sequence with 200 fMRI volumes per patient (repetition time/echo time = 3000/30 ms; flip angle = 90°; voxel size = 3.4 × 3.4 × 3.4 mm). PET data were acquired following intravenous injection of 18F‐labeled tracers: flortaucipir (6 × 5‐min frames, 75 to 105 min after injection), florbetapir (4 × 5‐min frames, 50 to 70 min after injection), and florbetaben (4 × 5‐min frames, 90 to 110 min after injection). A4/LEARN and ADNI imaging data were collected using congruent imaging protocols.

### 2.4 Image processing

All images were screened for artifacts before preprocessing, and processing was conducted independently for the ADNI and A4/LEARN samples. T1‐weighted structural MRI scans were bias‐corrected, segmented, and non‐linearly warped to Montreal Neurological Institute (MNI) space using the CAT12 toolbox. Dynamically acquired PET images were realigned and averaged to obtain single flortaucipir/florbetapir images, which were then rigidly registered to the T1‐weighted MRI scan. As reference regions, we used the inferior cerebellar gray for flortaucipir and whole cerebellum for florbetapir/florbetaben.^36^, ^37^ Reference regions and the cortical Schaefer atlas (200 regions of interest [ROIs]) were warped from MNI to T1‐native space using the CAT12‐derived non‐linear normalization parameters, masked with patient‐specific gray matter, and applied to PET data for determining SUVRs for each region of the Schaefer 200 atlas.^38^ Global florbetapir/florbetaben SUVRs for both cohorts (derived from identical cortical target regions for ADNI and A4/LEARN) were converted to Centiloids using equations provided by ADNI. To determine longitudinal tau‐PET changes, we used linear mixed models, including a fixed effect of time and subject‐specific random slopes and intercepts.^39^ Resting‐state fMRI images were preprocessed using an in‐house pipeline. Images were slice‐time corrected and realigned to the first volume for motion correction, followed by co‐registration to the respective T1‐weighted images. Using rigid‐transformation parameters, T1‐derived gray matter, eroded white matter, and eroded cerebrospinal fluid segments were transformed to EPI space. For denoising we regressed out nuisance covariates, including the time series of the eroded white matter and eroded cerebrospinal fluid and six motion parameters, as well as their time and dispersion derivatives and the global fMRI signal, detrending, and bandpass filtering (0.01 to 0.08 Hz) in native space. Global signal regression was applied to reduce global noise components and to enhance the specificity of functional connectivity estimates.^40^ To further reduce movement artifacts, we performed motion scrubbing, with volumes exceeding a 0.5‐mm framewise displacement threshold being removed, as well as the one prior and the two subsequent volumes. All included individuals had at least 5 min of resting‐state fMRI remaining after scrubbing.^41^ Spatial smoothing was not carried out to avoid artificially enhancing functional connectivity caused by signal spilling between adjacent brain regions. Preprocessed resting‐state fMRI images were subsequently warped to the MNI space using the CAT12‐derived spatial normalization parameters.

### 2.5 Connectivity analyses

After (pre)processing, functional images were parcellated via the 200 ROI Schaefer atlas^38^ analogous to the PET/diffusion data. Subsequently, functional connectivity was estimated as Fisher‐z‐transformed[Table alz71617-tbl-0001] Pearson correlation between ROI pairs. Individual matrices from 69 CN/Aβ− ADNI subjects were then averaged[Table alz71617-tbl-0002] to determine a connectivity template, which was used as the primary basis for the definition of functional hub regions. Structural connectivity matrices were generated from preprocessed diffusion MRI data from the 100 unrelated (young adult) subjects from the Human Connectome Project (HCP) 900 Subjects data release. These were corrected for susceptibility‐induced B0 field deviations, eddy current distortions, and subject motion and aligned to native structural space.^42^ For matrix reconstruction we applied a multi‐shell, multi‐tissue constrained spherical deconvolution tractography pipeline using tools from MRtrix3. In brief, this included T1‐weighted tissue segmentation to generate five tissue‐type tissue images, response function estimation (“dhollander” algorithm), estimation of the fiber orientation distribution, multi‐tissue informed log‐domain intensity normalization, modeling 10 million streamlines using anatomically constrained probabilistic streamlines tractography using dynamic seeding and cropping at the gray–white matter interface, and spherical deconvolution informed filtering of tractograms (“SIFT2” algorithm). Nodes of the connectome were defined according to the 200 ROI Schaefer parcellation (registered to diffusion space using Advanced Normalization Tools [ANTs]). Edges of the connectome were defined as the sum of SIFT2‐derived streamline weights between regions, as implemented in MRtrix3. Streamlines were assigned to nodes using a radial search of 3 mm to match the spatial resolution of the Schaefer parcellation, and each streamline's contribution to a given edge weight was additionally scaled by its length. SIFT2 filtering yields biologically plausible connectomes by ensuring that streamline density within each voxel is consistent with the fiber orientation distributions estimated from the diffusion‐weighted images.^43^ A group‐consensus binary network was then constructed with a method preserving density and edge‐length distributions of individual connectomes.^44^ Edges in the group consensus network were given weights through averaging the corresponding (log‐transformed) non‐zero edges across participants. Finally, weights were normalized to the range between zero and one.

### 2.6 Tau epicenter definition and node classification

Subject‐specific tau epicenters were defined as the 10 ROIs (i.e., 5%) with the highest baseline tau‐PET SUVRs, in line with our previous investigations (Figure S1).^45^, ^46^ Prior to the classification of regions into connectors or local nodes,^47^ regions were grouped into 17 a priori communities, following the spatial layout of established resting‐state networks.^29^ We then calculated the weighted degree (strength), within‐module degree z‐score, and normalized participation coefficient (NPC) for each node at different network densities (25%, 30%, and 35%), while ensuring overall network connectedness, before averaging them across densities to improve the robustness of the graph metrics.^33^, ^48^ To restrict the analysis to nodes with sufficient overall connectivity and within‐module prominence, only regions with a weighted degree above the 25th percentile and within‐module degree z‐score above one were considered for node classification.^49^ Connectors and local nodes were then identified based on NPC values, ensuring that nodes facilitating intermodular communication (connectors; high NPC) were distinguished from those with predominantly strong intramodular connectivity (local nodes; low NPC). This distinction was done for a range of percentile‐based splits of the NPC distribution (20/80%, 25/75%, and 30/70%), and the corresponding communication metrics (EBC, CME and WME) were calculated for each split (see “Efficiency calculations and null model”). To assess the robustness of node classification, we generated an independent functional connectivity template from CN participants in the human connectome aging (HCA) cohort (*n* = 83; age = 72 ± 8.1 years; 57.8% female; Montreal Cognitive Assessment ≥ 26). We evaluated the stability of node‐level graph measures (strength, within‐module degree z‐score, and NPC) across templates via Spearman correlation and used a non‐parametric spin test for inference. We also repeated the main analyses using a consensus node classification shared across templates. Mathematical descriptions for all employed graph measures, as well as results and information for the HCA data, can be found in the Supplementary Material.

### 2.7 Efficiency calculations and null model

Routing from epicenters was modeled based on the structural connectivity template, which involved the following steps to derive the shortest‐path efficiency for each connection: Strength‐to‐distance mapping for each edge (via inverse distance transform), calculation of ROI‐by‐ROI shortest‐path matrix (via Floyd‐Warshall algorithm), and squaring each off‐diagonal entry by −1.^33^ Combining our functional hub definition with this communication measure based on structural connectivity was motivated by the close coupling between these two complementary methods of defining brain connectivity^50^ and to avoid interpretational issues that arise from applying path‐based measures to functional brain networks.^33^ In this framework, functionally defined connector and local nodes serve as target regions, while structural connectivity defines the pathways along which communication efficiency from tau epicenters is quantified. EBC was then calculated as the difference between the average efficiency from a subject's tau epicenter set toward connectors (CME) and local nodes (WME), respectively. This was done separately for each NPC‐based split (see “Tau epicenter definition and node classification”). To enhance confidence in the specificity of our measures (EBC, CME, and WME), we repeated their calculation on a series of randomized versions of the original structural connectivity matrix, retaining its exact degree sequence and edge‐weight distribution, while also approximating its edge length distribution and overall weight‐length relationship.^51^ A total of 1000 such null networks were generated, and the derived null distributions were used to z‐normalize the empirical measures of each subject. Finally, these standardized values were averaged across splits, resulting in one EBC, CME, and WME value per subject, to be entered as predictors in the following robust regression analyses (see “Statistics”). While EBC constituted the primary predictor of interest, CME and WME were additionally analyzed in secondary models to disentangle the relative contributions of connectors and local nodes underlying EBC‐related effects on the outcomes of interest.

### 2.8 Modeling spatial tau spread and cognitive decline

To estimate the spatial extent of future tau accumulation at the individual level, we combined baseline regional tau‐PET SUVRs with ROI‐level tau change rates to extrapolate tau‐PET SUVRs 3 years from baseline, thereby standardizing follow‐up intervals across participants with varying longitudinal scan timings in ADNI. We then quantified spatial tau spread as the proportion of cortical parcels exceeding a predefined SUVR threshold of 1.3 in the extrapolated data. This measure captures the extent to which tau pathology disseminates across the cortex, rather than the magnitude of tau accumulation per se. A uniform threshold was applied across all regions to ensure that each parcel contributed equally to the extent metric and to preserve its interpretability as a global measure of spatial tau spread. In addition, spatial tau spread was recalculated for a range of SUVR thresholds to ensure robustness (see “Robustness and sensitivity analyses”). As a complementary analysis in ADNI, we also assessed spatial tau spread at the network level by quantifying the proportion of functionally defined modules showing enrichment of extrapolated tau accumulation, relative to spatially constrained null models generated via spin permutations (Supplementary Material). Subject‐level cognitive change rates were determined in (Aβ+) ADNI subjects, derived from repeated measurements of global cognition (MMSE), using linear mixed‐effects models with random intercepts and slopes. To increase sensitivity in the preclinical (Aβ+/CN) A4/LEARN cohort, we derived corresponding rates of change based on scores of the PACC.^52^

### 2.9 Statistical analysis

To assess associations between our dependent variables and EBC (as well as CME and WME), via its interaction with global Aβ levels (Centiloid), we used robust linear regression (Huber regression) to reduce the influence of outliers. Separate models were fit for each analysis, with covariates including age, sex, diagnosis at baseline (in the full ADNI sample), and the average Euclidean distance to individual tau epicenters (see “Robustness and sensitivity analyses”). *P* values for the Centiloid × EBC interaction were false discovery rate‐adjusted for the main analyses in our discovery dataset (ADNI).^53^

## 3 RESULTS

### 3.1 Node classification

Connectors were predominantly located within control, salience, and attentional networks, which are known to support cross‐network information integration,^54^ while local nodes were more evenly distributed across modules (Figure 2A). Connectors and local nodes did not differ significantly in their weighted degree distributions (two‐sample Kolmogorov‐Smirnov test: *p* = 0.26) and associated median values (Wilcoxon rank‐sum test: *p* = 0.19) pooled across splits. Cross‐template correspondence was satisfactory for our graph measures (average 𝜌 = 0.71, max *p* spin = 0.0001; Figure S2).

### 3.2 Higher epicenter broadcast capacity facilitates Aβ‐driven tau accumulation

**FIGURE 4:**
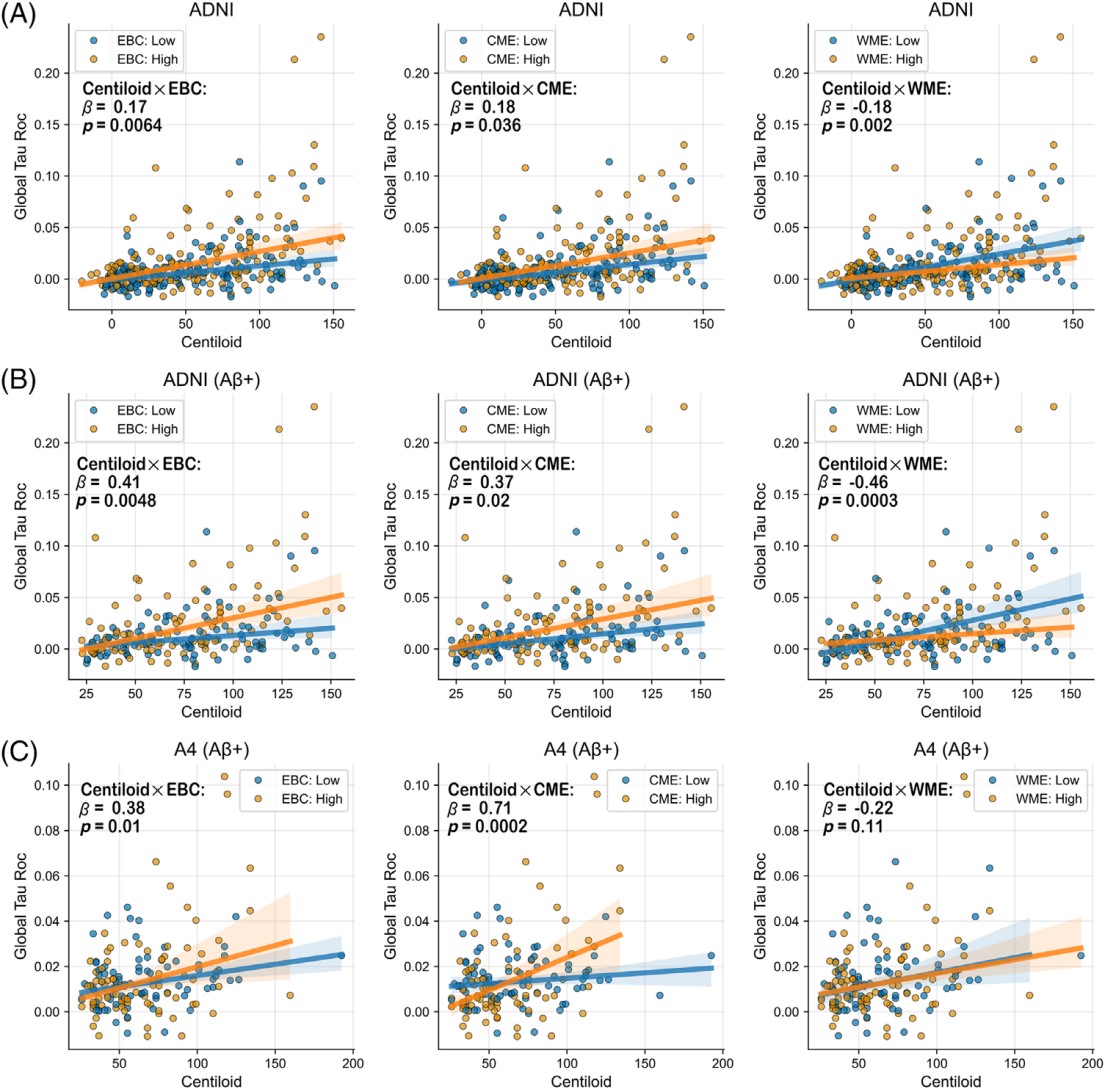
Epicenter broadcast capacity (EBC) modulates amyloid‐related tau accumulation rates. (A) Moderating effects of EBC (left), cross‐module efficiency (CME; middle), and within‐module efficiency (WME; right) on the relationship between baseline amyloid burden (Centiloid; *x*‐axis) and global tau accumulation rate (*y*‐axis) in ADNI. Higher EBC and CME are associated with higher amyloid‐related tau accumulation rates, whereas higher WME is associated with lower amyloid‐related tau accumulation rates. (B) Corresponding analyses restricted to amyloid‐positive (Aβ+) ADNI participants, showing the same pattern of results. (C) Corresponding analyses in preclinical A4/LEARN (Aβ+) participants. Higher EBC and CME are associated with steeper amyloid‐related increases in tau accumulation rates, whereas WME does not show significant moderation. Regression lines illustrate model‐predicted effects for low and high values of each metric (median split for visualization). Shaded bands indicate 95% confidence intervals. Reported *β* values are standardized regression coefficients from robust regression models. Roc,  rate of change.

To address our first aim, we assessed whether higher EBC was associated with faster Aβ‐related global tau accumulation. We tested the Centiloid × EBC interaction as a predictor of longitudinal global tau‐PET change, controlling for age, sex, and clinical diagnosis in the full ADNI sample. The Centiloid × EBC interaction was significant in ADNI (full sample: *β* = 0.17, *p* = 0.0064; Figure 4A; Aβ+ subjects only: *β* = 0.41, *p* = 0.0048; Figure 4B) and was replicated in the independent A4/LEARN cohort (*β* = 0.38, *p* = 0.01; Figure 4C). These results indicate that higher EBC amplifies the association between baseline amyloid burden and subsequent global tau accumulation. Examining the components of EBC, we observed a significant Centiloid × CME interaction in ADNI (full sample: *β* = 0.18, *p* = 0.036; Figure 4A; Aβ+ subjects only: *β* = 0.37, *p* = 0.02; Figure 4B) and in A4/LEARN (*β* = 0.71, *p* = 0.0002; Figure 4C), in line with the notion of efficient epicenter–connector communication contributing to enhanced amyloid‐related tau spread. In contrast, higher WME was associated with slower Aβ‐related tau accumulation, as reflected by significant Centiloid × WME interactions in ADNI (full sample: *β* = −0.18, *p* = 0.002; Figure 4A; Aβ+ subjects only: *β* = −0.46, *p* = 0.0003, Figure 4B), with preserved directionality in A4/LEARN (*β* = −0.22, *p* = 0.11; Figure 4C). Overall, these results suggest that amyloid‐driven future tau accumulation is accelerated when tau epicenters preferentially route to connectors but attenuated when routing is biased toward local nodes, consistent with opposing roles of broadcast versus containment pathways captured by EBC. Having established that EBC modulates the rate of amyloid‐related global tau accumulation as hypothesized, we next examined whether this effect extended to the spatial extent of downstream tau deposition, as predicted by the distinct topological roles of connectors and local nodes in shaping inter‐ versus intra‐network communication patterns.

### 3.3 Cross‐module efficiency modulates the spatial extent of Aβ‐related tau spread

**FIGURE 5:**
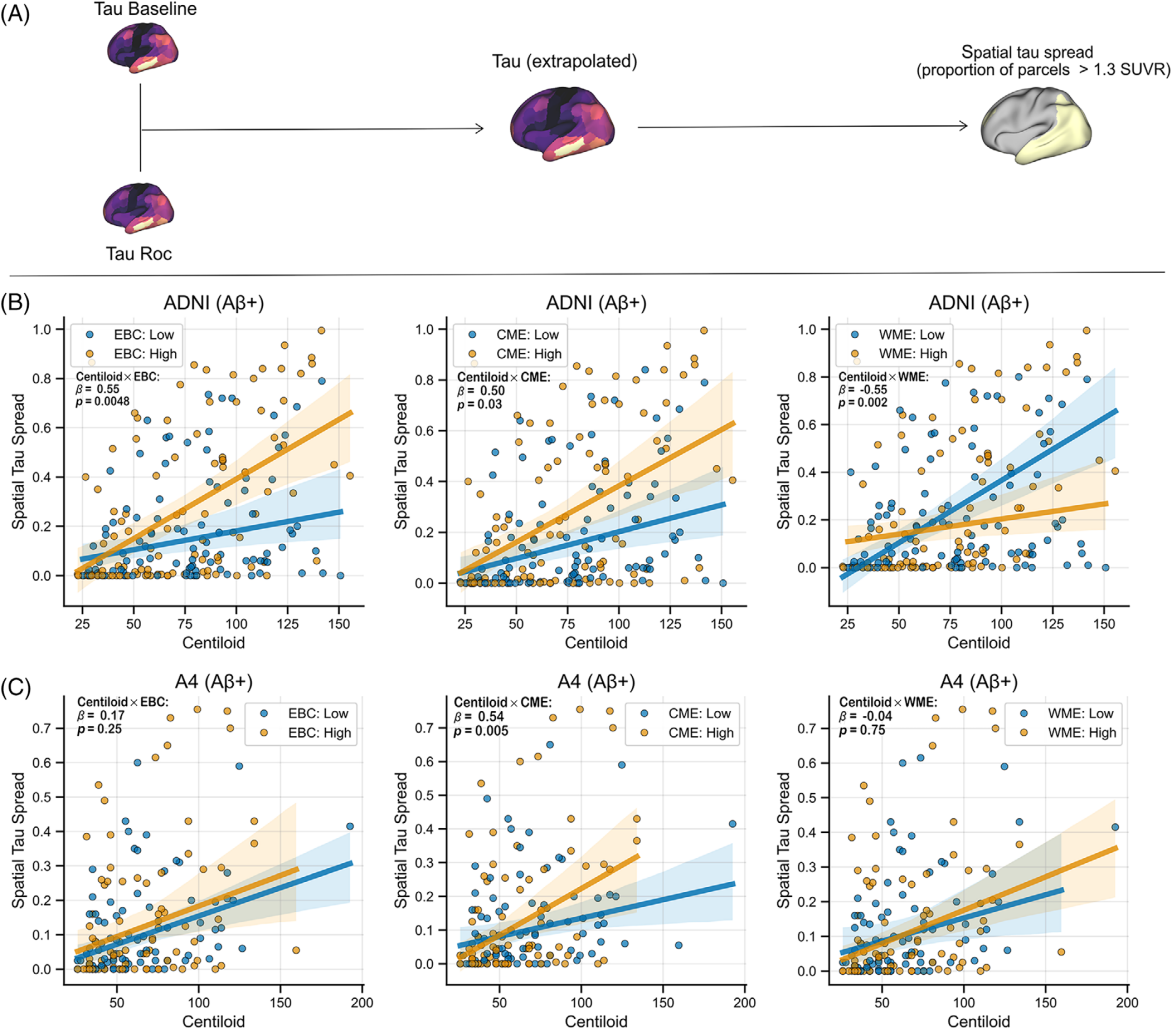
Epicenter broadcast capacity (EBC) modulates the spatial extent of amyloid‐related tau spread. (A) The spatial extent of downstream tau accumulation for a given subject is defined as the proportion of cortical parcels exceeding a tau‐PET SUVR threshold of 1.3 in extrapolated tau‐PET data. (B) Moderating effects of EBC (left), cross‐module efficiency (CME; middle), and within‐module efficiency (WME; right) on the relationship between baseline amyloid burden (Centiloid; *x*‐axis) and the spatial extent of downstream tau accumulation (spatial tau spread; *y*‐axis) in amyloid‐positive (Aβ+) ADNI participants. Higher EBC and CME are associated with greater spatial extents of amyloid‐related downstream tau accumulation, whereas higher WME is associated with reduced spatial extents. (C) Corresponding analyses in preclinical A4/LEARN (Aβ+) participants, where only higher CME is significantly associated with greater spatial extents of amyloid‐related downstream tau accumulation. Regression lines illustrate model‐predicted effects for low and high values of each metric (median split for visualization). Shaded bands indicate 95% confidence intervals. Reported *β* values are standardized regression coefficients from robust regression models. Aβ, amyloid beta; Roc, rate of change; SUVR, standardized uptake value ratio.

Our index of spatial tau spread (see “Modeling spatial tau spread and cognitive decline”) was entered as the dependent variable in a robust linear regression model testing the Centiloid × EBC interaction, controlling for age and sex (Figure 5A). These analyses were restricted to Aβ+ individuals. The Centiloid × EBC interaction was significant in ADNI (*β* = 0.55, *p* = 0.0048; Figure 5B), indicating that higher EBC amplified the association between baseline amyloid burden and the spatial extent of future tau deposition. This effect was not significant in the preclinical A4/LEARN cohort (*β* = 0.17, *p* = 0.25, Figure 5C). Decomposition of EBC revealed a significant Centiloid × CME interaction in both datasets (ADNI: *β* = 0.50, *p* = 0.03; Figure 5B; A4/LEARN: *β* = 0.54, *p* = 0.005; Figure 5C), indicating that more efficient communication between tau epicenters and connectors specifically predicts broader Aβ‐related spatial dissemination of cortical tau pathology. In contrast, higher WME was associated with a more limited spatial extent of Aβ‐related tau accumulation in ADNI (Centiloid × WME: *β* = −0.55, *p* = 0.002; Figure 5B), but not in A4/LEARN (Centiloid × WME: *β* = −0.04, *p* = 0.75; Figure 5C). This pattern is consistent with the attenuated Centiloid × EBC effect observed in the preclinical A4/LEARN cohort, given that EBC reflects the relative balance between CME and WME. Overall, these findings indicate that more efficient routing of tau epicenters to connectors promotes broader Aβ‐related spatial spread of tau pathology, whereas modulation by local node‐biased routing is reduced in the preclinical disease stage, resulting in a weaker moderating effect of EBC in A4/LEARN. Consistent with the parcel‐level results, higher CME amplified the association between baseline Aβ burden and cross‐module tau spread, supporting the notion that connectors act as key broadcasters for Aβ‐driven, inter‐network tau propagation (Figure S3). Finally, we addressed our third aim and tested whether higher EBC was linked to faster cognitive decline in patients on the AD spectrum (i.e. Aβ+), since faster EBC‐related tau spread should translate into faster cognitive deterioration.

### 3.4 Higher epicenter broadcast capacity accelerates Aβ‐driven cognitive decline

**FIGURE 6:**
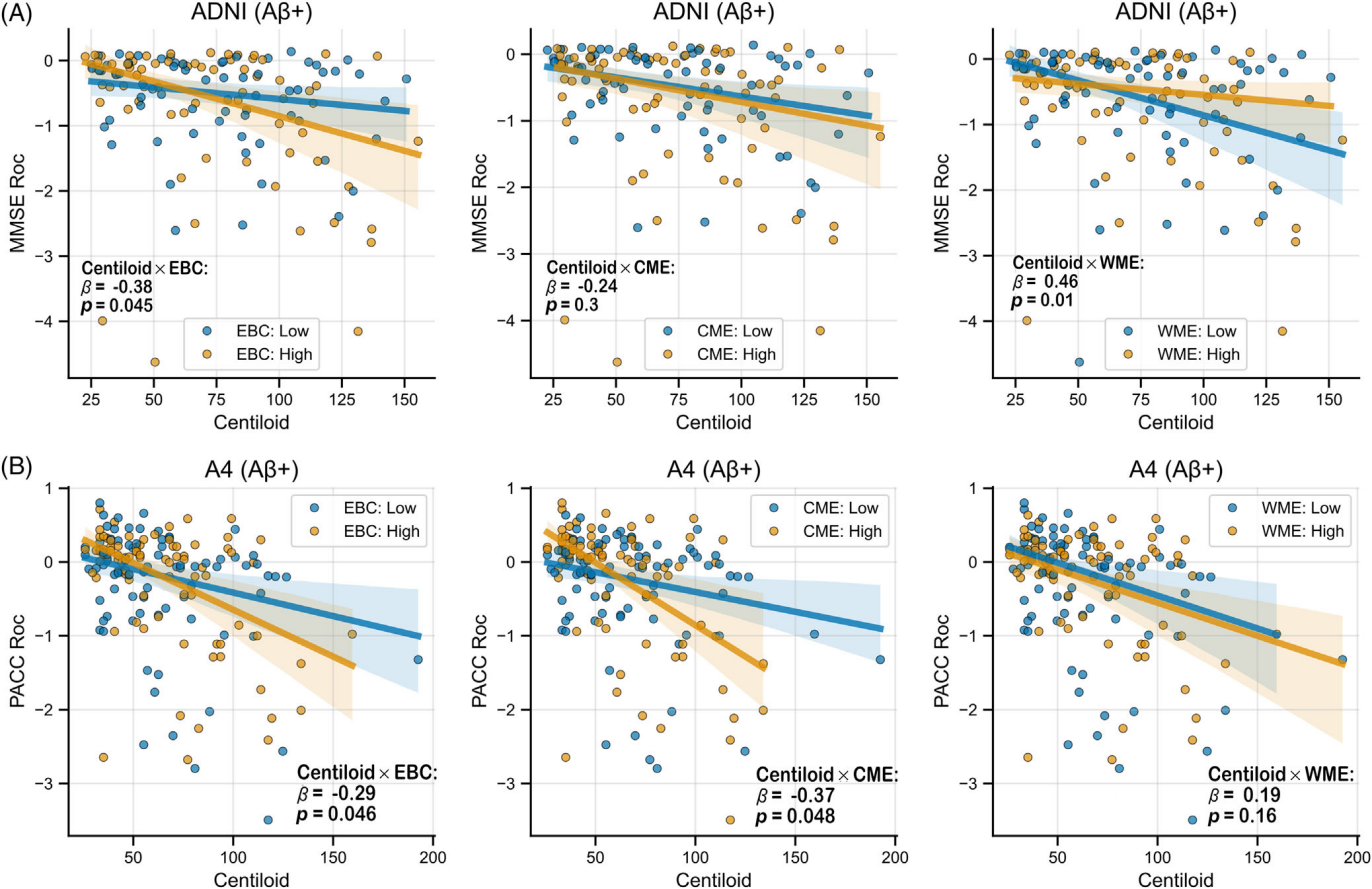
Epicenter broadcast capacity (EBC) modulates amyloid‐related cognitive decline. (A) Moderating effects of EBC (left), cross‐module efficiency (CME; middle), and within‐module efficiency (WME; right) on the relationship between baseline amyloid burden (Centiloid; *x*‐axis) and change rates in a global index of cognitive functioning (MMSE Roc; *y*‐axis) in amyloid‐positive (Aβ+) ADNI participants. Higher EBC and lower WME are associated with faster amyloid‐related cognitive decline, whereas CME does not show a significant moderating effect. (B) Corresponding analyses in preclinical A4/LEARN (Aβ+) participants: EBC and CME are associated with faster amyloid‐related cognitive decline, as indexed by change rates on the Preclinical Alzheimer's Cognitive Composite (PACC Roc; *y*‐axis), whereas WME does not show significant moderation. Regression lines illustrate model‐predicted effects for low and high values of each metric (median split for visualization). Shaded bands indicate 95% confidence intervals. Reported *β* values are standardized regression coefficients from robust regression models. Aβ, amyloid beta; MMSE, Mini‐Mental State Examination; Roc, rate of change.

To this end, robust regression models were fit with the Centiloid × EBC interaction as the predictor of cognitive change rates, controlling for age and sex. Supporting our hypothesis, we found a significant Centiloid × EBC interaction in ADNI (*β *= −0.38, *p *= 0.045; Figure 6A), which could be replicated in A4/LEARN (*β *= −0.29, *p *= 0.046, Figure 6B), suggesting that higher EBC relates to faster Aβ‐associated cognitive decline. Examination of the components underlying EBC revealed a differential pattern across cohorts. The Centiloid × CME interaction was not significant in ADNI (*β* = −0.24, *p* = 0.30; Figure 6A) but was significant in A4/LEARN (*β* = −0.37, *p* = 0.048, Figure 6B). Conversely, the Centiloid × WME interaction was significant in ADNI (*β* = 0.46, *p* = 0.01; Figure 6A) but not in A4/LEARN (*β* = 0.19, *p* = 0.16; Figure 6B). Together, these results indicate that while EBC robustly captures Aβ‐related cognitive decline across cohorts, the relative contributions of CME and WME differ by disease stage, with higher CME being more strongly associated with faster Aβ‐driven cognitive decline in preclinical AD (A4/LEARN) and higher WME being associated with slower decline at more advanced disease stages (ADNI; see “Discussion”).

### 3.5 Robustness and sensitivity analyses

To further assess the robustness of our findings, we performed a series of additional sensitivity analyses focusing on the main EBC‐related results in our primary dataset (ADNI). First, we repeated all primary analyses using a (consensus) node classification set derived from functional connectivity templates of the ADNI and HCA cohorts (Figure S4). This alternative node classification yielded results highly consistent with those of the main analyses (Figure S5). Second, although our network null model explicitly accounts for the spatial properties of brain connectivity (see “Efficiency calculations and null model”), we refit the EBC models while controlling for the difference in average Euclidean distance from tau epicenters to connectors and local nodes, which did not materially alter the direction or statistical significance of the Centiloid × EBC interaction for global tau accumulation rate (full sample: *β* = 0.15, *p* = 0.01; Aβ+ subjects only: *β* = 0.44, *p* = 0.001), spatial tau spread (*β* = 0.59, *p* = 0.001), or cognitive decline (*β* = −0.43, *p* = 0.02). In addition, to assess the robustness of spatial tau spread to the choice of SUVR threshold, we refit the corresponding models using lower cut‐offs for tau positivity (1.25 and 1.20 SUVR; Figure 5A). Across both alternative thresholds, the Centiloid × EBC interaction remained consistent in direction and significance (1.25 SUVR threshold: *β* = 0.44, *p* = 0.02; 1.20 SUVR threshold: *β* = 0.37, *p* = 0.05). Finally, excluding CN participants did not alter the direction or significance of the Centiloid × EBC interaction on spatial tau spread (*β* = 0.58, *p* = 0.03). Together, these sensitivity analyses indicate that the observed EBC‐related effects are robust to alternative node classifications, spatial distance controls, and threshold choices.

## 4 DISCUSSION

Our main finding is that the spread of tau pathology and the associated cognitive decline in AD are shaped by how tau epicenters are embedded within the brain's modular network architecture and by their access to brain regions that support cross‐network information broadcasting. Specifically, we found that more efficient communication of patient‐level tau epicenters with connectors relative to local nodes (higher EBC) amplified Aβ‐driven (i) global tau accumulation, (ii) large‐scale cortical tau dissemination, and (iii) cognitive decline. Across two independent and clinically heterogeneous AD cohorts (ADNI and A4/LEARN), these effects were most consistently driven by the efficiency of epicenter communication with connectors (CME), underscoring their central role in facilitating Aβ‐related tau propagation and its clinical consequences. Conversely, more efficient epicenter communication with local nodes (WME) was associated with the relative containment of tau spread and attenuated downstream effects, consistent with the proposed role of these regions in constraining cross‐network tau propagation (Figure 3). Together, these findings support a mechanistic model in which the topological position of tau epicenters relative to brain regions with distinct roles (captured by EBC) modulates Aβ‐driven tau propagation and clinical progression in AD. This highlights that the brain's network architecture and the emergence of tau pathology within this network are key determinants of individual variability in AD pathology and potential targets for network‐informed therapeutic strategies.^55^

Our first key finding was that more efficient routing of tau epicenters toward connectors, relative to local nodes (i.e., higher EBC), was linked to faster Aβ‐associated global tau accumulation across datasets (Figure 4), in line with the notion that the brain's network architecture acts as a scaffold that supports active tau dissemination from initial tau epicenters across the neocortex.^5^, ^46^ The present study extends this network‐based tau spreading concept by explicitly demonstrating that regions with distinct connectivity profiles differentially modulate the pace of Aβ‐driven tau accumulation. Global tau accumulation rates were consistently increased with higher CME, indicating a central role of connectors as amplifiers of brain‐wide tau propagation in the presence of cortical Aβ. This observation aligns with recent evidence of local and remote Aβ–tau interactions facilitating extensive neocortical tau spread in AD.^22^, ^45^, ^56^ In contrast, higher WME was associated with attenuated Aβ‐related global tau accumulation (Figure 4), suggesting that local nodes may confine tau spread to circumscribed networks, echoing our previous findings that higher brain network modularity limits tau dissemination and mitigates cognitive decline in AD.^31^, ^57^ Together, the current results sharpen the mechanistic understanding of how brain networks shape disease progression in AD, refining our prior observation that greater tau load in highly connected regions predicts earlier symptom onset.^58^ By highlighting the differential contributions of regions with different network signatures (captured by EBC), our findings substantially extend existing frameworks of AD pathology.

In our second finding, we showed that higher CME predicted more expansive Aβ‐related downstream tau deposition across multiple spatial scales (Figure 5B,C and Figure S3B), consistent with the intermodular connectivity profile of connectors. This result provides an important validation of our first aim, indicating that the EBC‐related effects on global tau accumulation rates at least partly reflect a broader Aβ‐driven spatial dissemination of tau, as anticipated by our theoretical framework (Figure 3). Although tau pathology generally follows a stereotypical spatial pattern in AD with advancing disease stage,^5^ there is substantial inter‐individual variability in the rate and extent to which tau spreads beyond initial epicenters.^1^, ^46^ Our findings indicate that this variability is systematically shaped by the interaction between Aβ burden and the efficiency of network communication between tau epicenters and connectors in association cortices. Interestingly, the attenuation of WME‐related effects in the preclinical A4/LEARN cohort, in contrast to the clinically more heterogeneous ADNI sample, suggests that early tau dissemination may depend primarily on epicenter access to cross‐module connectors, whereas local containment effects become more relevant at later disease stages. Consistent with this interpretation, tau deposition in connector regions is associated with an acceleratory phase of Aβ‐driven tauopathy in AD,^59^ marked by rapid spatial expansion of cortical tau.^56^ Given that the spatial extent of tau pathology is a key determinant of cognitive decline in AD,^60^, ^61^ these results provide a mechanistic link to our third finding that higher EBC predicts steeper Aβ‐associated cognitive decline (Figure 6). While this association was evident across cohorts and cognitive measures, the detrimental effects of high CME on cognition were more pronounced in the preclinical A4/LEARN cohort (Figure 6B) than in the more clinically advanced ADNI sample (Figure 6A). A possible explanation is that the PACC, used in A4/LEARN, is by design more sensitive to subtle deficits in higher‐order cognitive functions that depend heavily on proper connector functioning,^27^, ^49^, ^52^ whereas the MMSE primarily reflects global cognitive impairment and later‐stage decline.^2^, ^7^ In contrast, attenuating effects of elevated WME on cognitive decline were significant only in ADNI (Figure 6A), not in A4/LEARN (Figure 6B), suggesting that local node‐biased routing primarily constrains downstream effects once clinical impairment is already established. This may also reflect the fact that in preclinical AD, cognitive decline is not solely determined by the spatial extent of tau pathology but may also reflect early network‐level dysfunction associated with efficient access of tau epicenters to connectors in the presence of Aβ. Together, these results emphasize that EBC captures distinct underlying components whose relevance may differ across disease stages.

When interpreting the findings of our study, several limitations should be considered. First, since the tau‐PET tracer flortaucipir exhibits substantial off‐target binding to subcortical structures unrelated to tau,^62^ we decided to exclude these regions from our analysis, possibly resulting in significant loss of information, since subcortical brain regions contribute to the brain's modular architecture.^63^, ^64^ Here, future investigations with second‐generation tau‐PET tracers that show a better off‐target binding profile will be key for the replication and extension of our results. Second, we employed a template‐based approach for the derivation of EBC (Figure 2) to isolate network‐constrained propagation mechanisms from the effects of neurodegeneration. This was further motivated by the limited amounts of harmonized high‐resolution subject‐level fMRI data in both samples and the absence of diffusion MRI data in the replication dataset (A4/LEARN). Defining node roles via group‐average data is not uncommon, and our main results were robust across templates (Figure S5),^30^, ^33^ yet individualized approaches should be considered in the future and may allow more nuanced conclusions about how individual brain network topology shapes tau spreading patterns across the AD spectrum, with node roles shifting due to network reorganization.^65^ Similarly, our approach is agnostic to patient‐specific changes in white matter anatomy often prevalent in AD^66^, ^67^ but should provide a realistic anatomical framework for modeling network‐based tau spreading in AD.^68^ Finally, EBC was based on the established shortest‐path routing mechanism,^69^ but alternative communication models could be explored in future work.^70^

In conclusion, we provide compelling in vivo evidence that the network embedding of individual tau epicenters in the brain's modular network architecture critically shapes Aβ‐driven effects on spatiotemporal tau dynamics and clinical trajectories in AD. We show that tau epicenter patterns that allow relatively more efficient network communication with functional connector regions facilitate the Aβ‐related spread of tau pathology and cognitive decline. These findings align with the amyloid cascade model of AD and the proposition that Aβ facilitates cortical tau dissemination along the scaffold of the brain's network architecture.^4^, ^15^, ^23^ Our topological perspective highlights the potential of connectome‐based biomarkers for improving risk stratification and disease monitoring in AD,^46^, ^58^ while pointing to connectors as potential targets for future interventions aimed at limiting tau pathology spread. Since tau pathology in AD is influenced by multiple factors besides network topology including co‐pathologies^71^, ^72^, regional myelination,^73^ and apolipoprotein E ε4 carriership,^74^, ^75^ integrating our current approach with these aspects represents an exciting avenue for future research and should yield even deeper insights into the pathophysiological mechanisms of AD.

## CONFLICT OF INTEREST STATEMENT

Fabian Hirsch has nothing to disclose. Lukas Frontzkowski has nothing to disclose. Anna Steward has nothing to disclose. Sebastian N. Roemer‐Cassiano has nothing to disclose. Davina Biel has nothing to disclose. Zeyu Zhu has nothing to disclose. Carla Palleis has nothing to disclose. Johannes Gnörich has nothing to disclose. Madleen Klonowski has nothing to disclose. Günter Höglinger participated in industry‐sponsored research projects from Abbvie, Amylyx, Biogen, Biohaven, Ferrer, Novartis, Roche, Sanofi, Teva, Takeda, and UCB; has ongoing research collaborations with Roche, UCB, and Abbvie; and serves as a consultant for Abbvie, Alzprotect, Amylyx, Aprinoia, Asceneuron, Bayer, Bial, Biogen, Biohaven, Epidarex, Ferrer, Kyowa Kirin, Lundbeck, Novartis, Retrotope, Roche, Sanofi, Servier, Takeda, Teva, and UCB. Günter Höglinger received honoraria for scientific presentations from Abbvie, Bayer, Bial, Biogen, Bristol Myers Squibb, Esteve, Kyowa Kirin, Pfizer, Roche, Teva, UCB, and Zambon. Günter Höglinger holds two patents (G. U. Höglinger, M. Höllerhage, T. Rösler, “Treatment of synucleinopathies,” US Patent no.: US 10,918,628 B2, date of patent: Feb. 16, 2021; and G. U. Höglinger, M. Höllerhage, T. Rösler, “Treatment of synucleinopathies,” European Patent no.: EP 17 787 904.6‐1109/3 525 788). Matthias Brendel is a member of the Neuroimaging Committee of the EANM. Matthias Brendel has received speaker honoraria from Roche, GE Healthcare, Iba, and Life Molecular Imaging; has advised Life Molecular Imaging and GE Healthcare; and is currently on the advisory board of MIAC. Nicolai Franzmeier has nothing to disclose. Author disclosures are available in the Supporting Information.

## CONSENT STATEMENT

All study participants whose data are used in this study provided written informed consent.

## Supporting information




**Supporting Information**: alz71617‐sup‐0001‐SuppMat.docx

**Supporting Information**: alz71617‐sup‐0002‐SuppMat.pdf
