# Supplementary material for: Modular brain networks shape amyloid‐driven tau spread and cognitive decline
Source: Alzheimers Dement. 2026 Jul 8;22(7):e71617. doi: 10.1002/alz.71617 (PMC13344889; doi:10.1002/alz.71617)
Supplement: Supplementary file 1 — Supporting Information: alz71617‐sup‐0001‐SuppMat.docx [file ALZ-22-e71617-s002.docx]

Supplement to: *Modular brain networks shape amyloid-driven tau spread and cognitive decline*

**Authors:** Fabian Hirsch^a^, Lukas Frontzkowski^a,b^, Anna Steward^a^, Sebastian N. Roemer-Cassiano^a,c,d,e^, Davina Biel^a^, Zeyu Zhu^a^, Carla Palleis^c,f,g^, Johannes Gnörich^b^, Madleen Klonowski^a^, Günter Höglinger^c,f,g^, Matthias Brendel^b,f,g†^, Nicolai Franzmeier^a,g,h*†^

**Affiliations:**

^a^Institute for Stroke and Dementia Research (ISD), University Hospital, LMU Munich, Munich, Germany

^b^Department of Nuclear Medicine, University Hospital, LMU Munich, Munich, Germany

^c^Department of Neurology, University Hospital, LMU Munich, Munich, Germany

^d^Max Planck School of Cognition, Leipzig, Germany

^e^Department of Clinical Neurosciences, University of Cambridge School of Clinical Medicine, Cambridge, UK

^f^German Center for Neurodegenerative Diseases (DZNE), Munich, Germany

^g^Munich Cluster for Systems Neurology (SyNergy), Munich, Germany

^h^University of Gothenburg, The Sahlgrenska Academy, Institute of Neuroscience and Physiology, Department of Psychiatry and Neurochemistry, Gothenburg, Sweden

*Corresponding author

Email: [Nicolai.Franzmeier@med.uni-muenchen.de](mailto:Nicolai.Franzmeier@med.uni-muenchen.de)

† These authors contributed equally to this work.

*HCA data:*

Extensively preprocessed and denoised fMRI data (*N* = 172; age-range: 59-89 years; 54.7% female) from the HCP-Aging (HCA) project were downloaded from <https://balsa.wustl.edu/> (details about the preprocessing pipelines can be found here https://www.humanconnectome.org/study/hcp-lifespan-aging/project-protocol/imaging-protocols-hcp-aging). We then performed global signal regression [1], before parcellating the functional images with the 200 ROI Schaefer atlas [2], followed by band-pass filtering (0.01 to 0.08 Hz). Functional connectivity matrices were calculated via pairwise correlations of the regional timeseries. Subsequently, matrices from cognitively normal subjects (MOCA > 26; *n* = 83) were Fisher-transformed and averaged to derive the template. Cross-template correspondence of the relevant node-wise graph-measures was assessed (Figure S2), and node classification was done as described in the main manuscript (see ‘Methods: Tau epicenter definition and node classification’).

*Graph measures for hub derivation:*

The weighted degree $k_{i}$ (strength) for a node $i$ represents the sum of weights of all connections associated with that node [3]:

$$k_{i}=\sum_{j\in N} w_{ij}$$

where $w_{ij}$is the weight of the connection between nodes $i$ and $j$, and $N$ represents the number of nodes within the network. The within-module degree z-score quantifies how strongly connected node $i$ is relative to other nodes within its own module $m_{i}$ [4]:

$$z_{i}=\frac{k_{i}\left( m_{i} \right)-\bar{k}\left( m_{i} \right)}{\sigma^{k\left( m_{i} \right)}}$$

where $k_{i}\left( m_{i} \right)$ is the weighted degree of node $i$ within its module $m_{i}$, $\bar{k}\left( m_{i} \right)$ is the mean weighted degree within module $m_{i}$, and $\sigma^{k\left( m_{i} \right)}$ is the standard deviation of weighted degrees within module $m_{i}$. The normalized participation coefficient measures the diversity of a node's connections strengths across network modules, while adjusting for expected intramodular connectivity [5]:

$$\text{PC}_{\text{norm}_{i}}=1-\sqrt{B_{0}\sum_{m\in M} \left( \frac{k_{i}\left( m \right)-k_{i}\left( m \right)_{\text{rand}}}{k_{i}} \right)^{2}}$$

where $k_{i}\left( m \right)$ denotes the weighted degree of node $i$ within module $m$, $k_{i}\left( m \right)_{\text{rand}}$ is the median intramodular weighted degree of node $i$ across a series of degree- and strength preserving null networks, and $k_{i}$ is the (total) weighted degree of node $i$. The multiplicative term $B_{0}$ constrains the range of $\text{PC}_{\text{norm}}$ to between 0 and 1 and was set to 0.5 in our study.

*Consensus node set:*

A consensus node set was defined as the intersection of connectors and local nodes respectively, in the ADNI and HCA templates at the least conservative NPC-split (see ‘Methods: Tau epicenter definition and node classification’). Epicenter broadcast capacity (EBC) was then calculated as described in the main text (see ‘Methods: Efficiency calculations and null model’) and used to replicate our main results from the ADNI dataset (Fig. S4 and Fig. S5).

*Figures:*


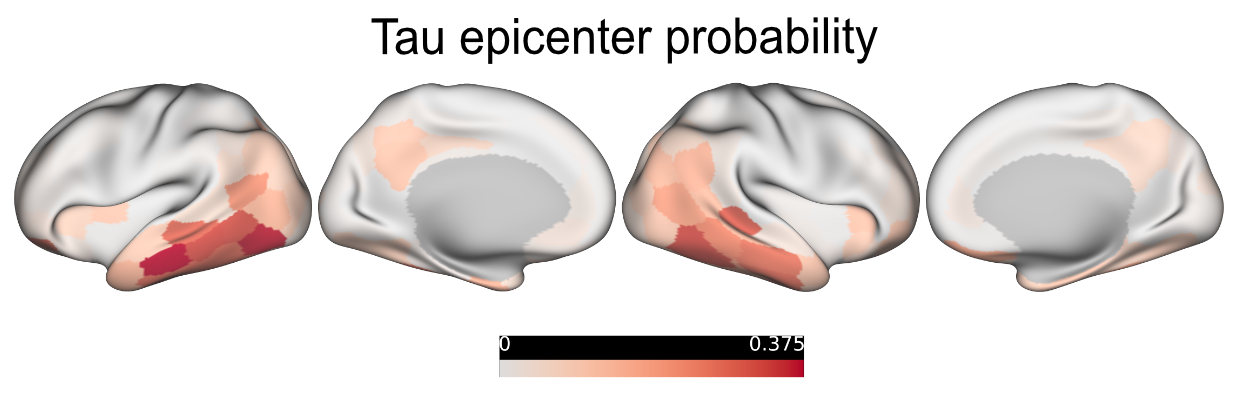


**Fig. S1. Spatial distribution of epicenter probability across ADNI subjects.** Subject-specific tau epicenters were defined as the 10 regions with highest baseline tau-PET SUVR. Epicenter probability was calculated for each region as the percentage of subjects for whom that region belonged to the epicenter set. SUVR = standardized uptake value ratio.


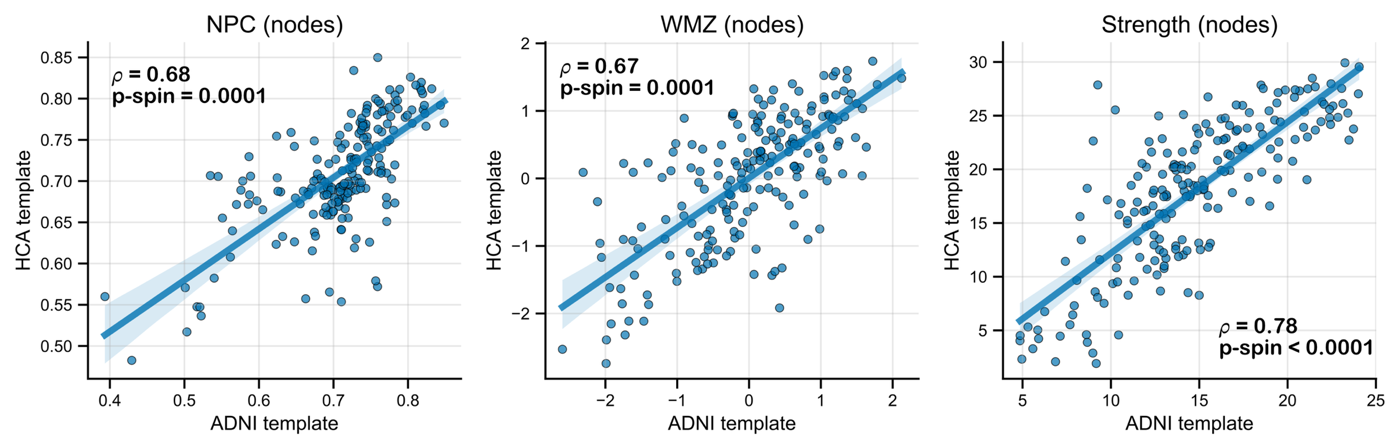


**Fig. S2. Correspondence of node-level graph metrics between ADNI and HCA functional connectivity templates.** Correspondence between node-wise graph measures derived from ADNI- (x-axis) and Human Connectome Project Aging (HCA; y-axis) functional connectivity templates for normalized participation coefficient (NPC; left), within-module z-score (WMZ; middle), and node strength (right). Solid lines indicate linear fits; shaded bands represent 95% confidence intervals. Reported ρ values denote Spearman rank correlations; p-values were derived from spin-based permutation tests accounting for spatial autocorrelation [6].


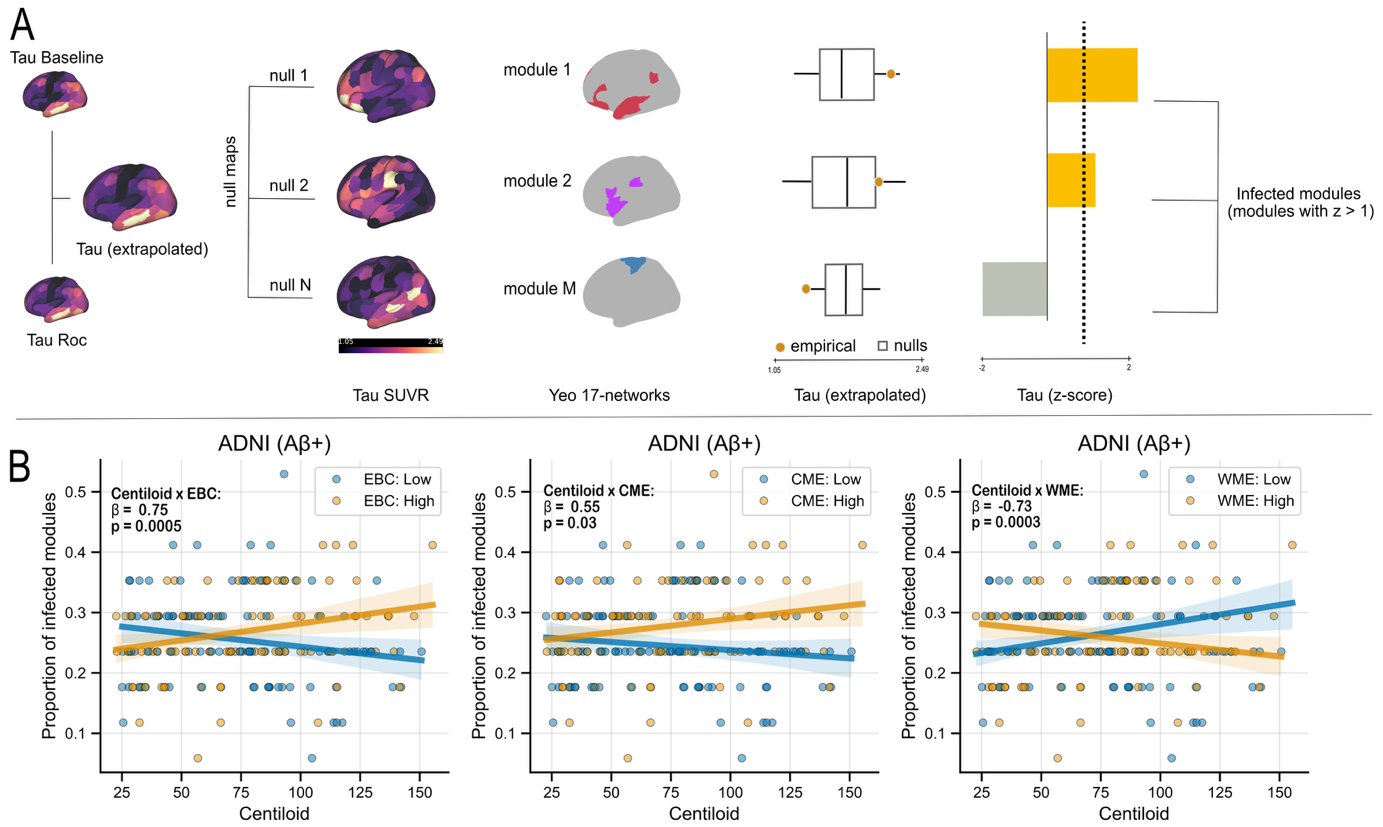


**Fig. S3.** **Epicenter broadcast capacity (EBC) modulates amyloid-related downstream tau spread across functional network modules.** **(A)** Derivation of module-level downstream tau spread. Subject-specific extrapolated tau-PET maps were compared against spatially constrained null models (spin permutations) and subsequently down-sampled to the scale of 17 canonical resting-state networks. Network modules were classified as “infected” if their empirical extrapolated tau signal exceeded the null distribution (z > 1), yielding for each subject the proportion of infected functional modules. **(B)** Moderating effects of EBC (left), cross-module efficiency (CME; middle), and within-module efficiency (WME; right) on the relationship between baseline amyloid burden (Centiloid; x-axis) and the proportion of infected functional modules (y-axis) in amyloid-positive (Aβ+) ADNI participants. Regression lines illustrate model-predicted effects for low and high values of each metric (median split for visualization); shaded bands indicate 95% confidence intervals. Reported β-values correspond to standardized regression coefficients from robust regression models. Aβ = amyloid-β; SUVR = standardized uptake value ratio.


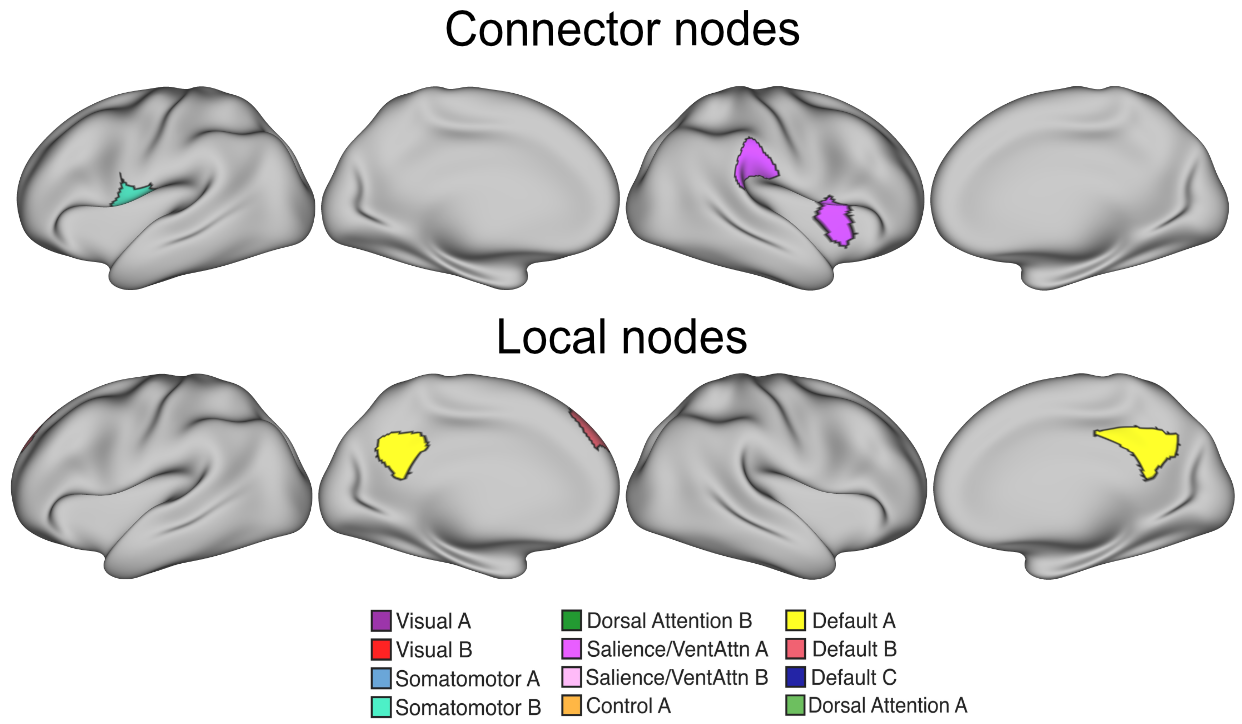


**Fig. S4. Consensus node set.** Spatial distribution of functional connector and local nodes shared across ADNI- and HCA functional connectivity templates, color-coded by canonical resting-state networks. HCA = Human Connectome Project Aging.


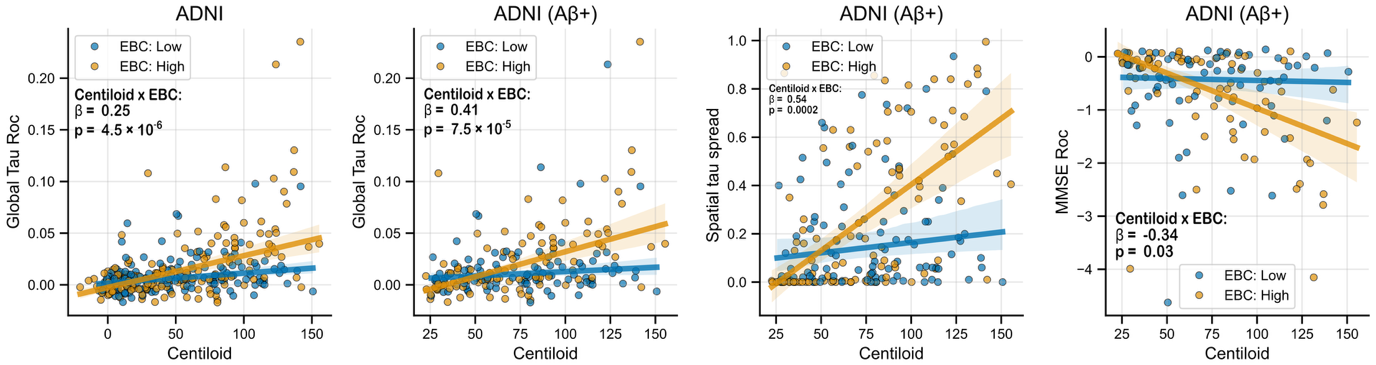


**Fig. S5. Replication of main results using a consensus node classification set shared across ADNI and HCA functional connectivity templates.** Moderating effects of epicenter broadcast capacity (EBC) on the relationship between baseline amyloid burden (Centiloid; x-axis) and global tau accumulation rate (Global Tau Roc; y-axis) are shown for the full ADNI sample (left) and for amyloid-positive (Aβ+) participants only (left-middle). Additional panels depict moderating effects of EBC on the spatial extent of downstream tau accumulation (Spatial Tau Spread; right middle) and on cognitive decline (MMSE Roc; right) in Aβ+ ADNI participants. Regression lines illustrate model-predicted effects for low and high EBC values (median split for visualization); shaded bands represent 95% confidence intervals. Reported β-values correspond to standardized regression coefficients from robust regression models. Aβ = amyloid-β; MMSE = Mini Mental Status Examination; Roc = rate of change.

*References:*

1. Burgess, G.C., et al., *Evaluation of Denoising Strategies to Address Motion-Correlated Artifacts in Resting-State Functional Magnetic Resonance Imaging Data from the Human Connectome Project.* Brain Connect, 2016. **6**(9): p. 669-680.

2. Schaefer, A., et al., *Local-Global Parcellation of the Human Cerebral Cortex from Intrinsic Functional Connectivity MRI.* Cereb Cortex, 2018. **28**(9): p. 3095-3114.

3. Rubinov, M. and O. Sporns, *Complex network measures of brain connectivity: Uses and interpretations.* NeuroImage, 2010. **52**(3): p. 1059-1069.

4. Guimerà, R. and L.A. Amaral, *Cartography of complex networks: modules and universal roles.* J Stat Mech, 2005. **2005**(P02001): p. nihpa35573.

5. Pedersen, M., et al., *Reducing the influence of intramodular connectivity in participation coefficient.* Network Neuroscience, 2020. **4**(2): p. 416-431.

6. Markello, R.D. and B. Misic, *Comparing spatial null models for brain maps.* NeuroImage, 2021. **236**: p. 118052.
